# Sex-related inequalities in crude and age-standardized suicide rates: trends in Ghana from 2000 to 2019

**DOI:** 10.1186/s12889-024-18516-8

**Published:** 2024-04-17

**Authors:** Faustina Adoboi, Aliu Mohammed, Precious Adade Duodu, Richard Gyan Aboagye, Abdul-Aziz Seidu, Florence Gyembuzie Wongnaah, Bright Opoku Ahinkorah

**Affiliations:** 1 Cape Coast Nursing and Midwifery Training College, Cape Coast, Ghana; 2 Department of Health, Physical Education, and Recreation, University of Cape Coast, Cape Coast, Ghana; 3 Department of Nursing, School of Human and Health Sciences, University of Huddersfield, Queensgate, Huddersfield, England, UK; 4 School of Population Health, Faculty of Medicine and Health, University of New South Wales, 2052 Sydney, NSW Australia; 5 Department of Family and Community Health, Fred N. Binka School of Public Health, University of Health and Allied Sciences, Hohoe, Ghana; 6 College of Public Health, Medical and Veterinary Sciences, James Cook University, Townsville, 4811 Queensland, QLD Australia; 7 Centre for Gender and Advocacy, Takoradi Technical University, Takoradi, P.O. Box 256, Ghana; 8 REMS Consultancy Services Limited, Sekondi-Takoradi, Western Region Ghana; 9 Department of Global Public Health, Karolinska Institutet, Stockholm, Sweden; 10 School of Clinical Medicine, University of New South Wales Sydney, Sydney, Australia

**Keywords:** Sex differences, Suicide, Crude, Age-standardized, Inequality, Ghana

## Abstract

**Background:**

Suicide represents a major public health concern, affecting a significant portion of individuals. However, there remains a gap in understanding the age and sex disparities in the occurrence of suicide. Therefore, this study aimed to investigate the sex-related inequalities in suicide rates in Ghana from 2000 to 2019.

**Methods:**

We utilized data from the WHO Health Equity Assessment Toolkit (HEAT) online software. We analysed sex differences in both crude and age-standardized suicide rates in Ghana spanning from 2000 to 2019. Crude and age-adjusted suicide rates were calculated based on the International Classification of Diseases (ICD) definition and coding of suicide mortality. We measured inequality in terms of sex. Two inequality indicators were used to examine the suicide rates: the difference (D) and the ratio (R).

**Results:**

Age-standardized and crude suicide rates in Ghana were higher among men from 2000 to 2019. Between 2000 and 2007, the age-standardized suicide rate for women rose steadily and declined slightly between 2008 and 2019. Age-standardized suicide rates for men increased consistently from 2000 to 2010, then declined steadily from 2011 to 2019. The crude suicide rates among men and women followed similar patterns. The widest absolute inequality in crude suicide rates (D) was recorded in 2013 (D=-11.91), while the smallest difference was observed in 2000 (D=-7.16). We also found the greatest disparity in age-standardized rates in 2011 (D=-21.46) and the least in 2000 (D=-14.32). The crude suicide rates increased with age for both men and women aged 15–54 years and 55–85+ years respectively. However, the increased rate was higher in men than in women across all age groups surveyed. A similar pattern was observed for relative inequality in both crude and age-standardized rates of suicide.

**Conclusion:**

The suicide rate in Ghana has declined over time. Suicide is more common among older men. Inequalities in suicide rates, in both absolute and relative terms, are similar. There is a need to monitor suicide trends in Ghana, especially among older men. Moreover, the findings could serve as a basis for future studies on suicide in Ghana.

## Introduction

Suicide is a major public health concern necessitating urgent interventions. It is characterized by death caused by self-directed injurious behaviour with intent to die as a result of the behaviour [1, 2]. Globally, several efforts have been made to curb the phenomenon [3–5]. However, suicide accounts for over 703,000 deaths annually (one in every 100 deaths), representing 1.3% of all deaths in 2019, although more than this number attempted suicide [6–8]. The commonest methods of suicide include pesticide ingestion, hanging, and firearms. Pesticide self-poisoning alone contributes to about 20% of global suicides, most of which occur in rural agricultural areas in low-and middle-income countries (LMICs) [6]. The tragedy has tremendous long-lasting effects on families, communities, and countries [6].

Although suicide affects all global regions, LMICs including Ghana, disproportionately accounted for over 77% of global suicides (703,000) in 2019 [6]. In the same year, the global suicide rate average (9.0 per 100,000) was lower compared to that of the World Health Organization (WHO) African region (11.2 per 100,000) [7]. In Ghana, 6.6 individuals per 100,000 people (177 females and 1816 males) in 2019, or an estimated 1,500 persons annually in the general population committed suicide [9]. Until March 2023, suicide was criminalized in Ghana and those who attempted it were prosecuted [10–13]. However, attempted suicide is now decriminalized or depenalized in Ghana following a recent repeal of the statutes criminalizing non-fatal suicidal behaviour [12]. Significantly, Ghana is faced with an acute shortage of mental health professionals and underfunding of mental healthcare services [14–16].

Suicide is a complicated problem that is influenced by a variety of factors including demographics, socioeconomic level, sociocultural status, mental illness, history of suicidal behaviours, and the effectiveness of healthcare systems in preventing suicide [17–19]. Other factors that could lead to suicide are loss of economic control or financial difficulties, hopelessness, sexual incompetence, break-ups from relationships, chronic pain, and illness amongst others [20–22]. Suicide is more prevalent in unmarried persons [23], the unemployed [24], individuals in lower socioeconomic groups [25], and people in traumatic situations [26]. To this end, protective factors such as healthy coping skills, supportive social interactions, safe community environments, and affordable healthcare [19, 27] constitute some of the timely, evidence-based, and low-cost interventions for suicide prevention.

Sex-related disparities in suicidal behaviour are partially attributed to emotional and behavioural issues [28]. The male-to-female suicide ratio increases with age, particularly in older adolescents, often linked to diagnosed mental illness [29–32]. In 2021, suicide ranked among the top 9 causes of death for individuals aged 10 to 64 years, ranking as the second-leading cause for ages 10–14 yearsand 20–34 years, and the fourth-leading cause for those aged 15–29 years in both sexes [2]. Men face a notably elevated suicide rates within Africa and globally [4, 33].

A recent Ghanaian study showed that suicide rates between the years 2000 and 2015 involved more males than females with the main reason being lack of financial resources [20]. In Ghana, both fatal and non-fatal suicidal behaviours are predominantly male problems with multiple nuances [34–36]. Abdulai’s [1] analysis of the trends of 142 online news media reports of suicides found that the victims of suicides were predominantly males (85.92%), and young (mean age = 34.81 ± 15.71 years; range 10–86 years). Suicide by hanging (67.94%), the use of firearms (18.32%), and self-poisoning (8.93%) were the common methods used by the victims [1]. A retrospective review of coroners’ reports of 309 decedents over a decade (2008 to 2017) within the northern part of Ghana found that approximately 61% were males, with ages ranging from 5 to 81 years [37]. Also, suicide was highest among the aged 30–39 years group with hanging and poisoning being the most common methods employed. Stigmatization and psychosocial problems arising from chronic illness and economic hardships were significant triggers of suicide [37]. Conversely, systematic reviews and meta-analyses have found the rate of suicide to be three times higher for women than men after release from incarceration. Similarly, Janca et al. [26] and Miranda-Mendizabal et al. [38] also found females to present a higher risk of suicide attempts.

There is a paucity of literature on suicide in Africa [39, 40]. Although suicide-related research in Ghana has progressed over the last decade, it appears to focus more on the adult population [41]. Few Ghanaian studies have focused on suicide in younger generations. These studies only focused on the probable causes of suicide but not the inequalities related to the sex and age in the country [41–44]. This study aimed to fill the gap by examining the sex differences in crude and age-adjusted suicide rates from 2000 to 2019 in Ghana using data from the WHO’s Health Equity Assessment Toolkit (HEAT). Understanding these inequalities can be effective in developing and implementing suicide prevention programmes, especially as suicide and its mortality rates are indicators of target 3.4 of the Sustainable Development Goals (SDGs) [45].

## Methods

### Data source

Data for this study was sourced from the WHO Health Inequality Data Repository, which can be accessed at the Global Heath Observatory (GHO) platform via https://www.who.int/data/gho/data/themes/mental-health/suicide-rates [46]. The GHO is WHO’s data repository for health-related statistics for its 194 Member States, with over 1000 indicators on priority health issues such as child mortality, maternal mortality, and mortality due to non-communicable diseases, suicide, pollution, road traffic injuries, homicide, natural disasters, and conflict [8]. In this study, the detailed data sources and methodology are available elsewhere [8, 47]. Briefly, all-cause mortality rates by age and sex for WHO Member States were derived from life tables which draw on United Nations (UN) World Population Prospects 2015 revision. The specific-cause mortality was based on WHO and UN Interagency estimation processes, which made use of epidemiological studies, disease registers and notifications systems whilst other causes of death for populations without useable death-registration data were determined using the Global Burden of Diseases (GBD) estimation process [48]. These WHO Global Health Estimates (GHE) give a complete and comparable collection of cause-of-death estimates from 2000 onwards, consistent with and including estimates from United Nations agencies, interagency organizations, and WHO for population, births, all-cause deaths, and specific causes of death [49].

### Measures

Crude and age-standardized suicide rates was the outcome measure of interest. Crude suicide rate was computed as the number of suicides per 100,000 population. The suicide rate was estimated using the GBD study protocol, which utilized the International Classification of Diseases (ICD) definition and coding of suicide mortality [8, 50]. The specific ICD-10 codes used were ICD-10 × 60–X84, and Y87. 0. This estimate was incorporated into the WHO GHE and available for use from the WHO Health Inequality Data Repository. We only conducted the analysis using the estimated data available online through the WHO HEAT software [51].

Sex was the inequality dimension used in our study. Data on suicide rates from the WHO Health Inequality Data Repository was available for disaggregation by cause, sex, and age only (https://www.who.int/data/gho/data/themes/mental-health/suicide-rates). However, for Ghana, only sex segregation was available. Sex was grouped into male and female.

### Statistical analysis

The WHO HEAT online software version 3.1 [51] was used for this study’s analysis. The WHO HEAT is an online statistical tool for examining health disparities within and between nations on several health and social issues such as indicators of morbidities and mortalities, with a detailed description of the software and its usage available in the literature [51]. We examined the suicide rate across sex using two inequality indicators. The difference (D) and the ratio (R) were these two measures of inequality. R is a relative measure, whereas D is an absolute summary measure. D is an absolute measure of inequality that indicates the difference between two population subgroups. D was calculated by deducting the suicide rate in males from females for each year from 2000 to 2019. For inequality to exist, D takes on a value greater than zero. Greater absolute values of D indicate higher inequality in suicide rates whereas zero value showed no evidence of inequality. Hence, high negative value of D signify inequality among the males while high positive value shows inequality among the females. Alternatively, R was estimated as the ratio of two distinct subpopulations. In this study, R was calculated as R = Y_female_/Y_male_. For no inequality in suicide rate to exist, R takes the value of one. R takes only positive values. The further the value of an R from one, the higher the level of inequality. Further information on the calculation of these measures —D and R—have been highlighted in the literature [51, 52].

### Ethical consideration

No ethical clearance was sought for this study because the dataset is available in the public domain. However, permission to use the WHO HEAT software was granted by the WHO upon request.

## Results

**Fig. 1:**
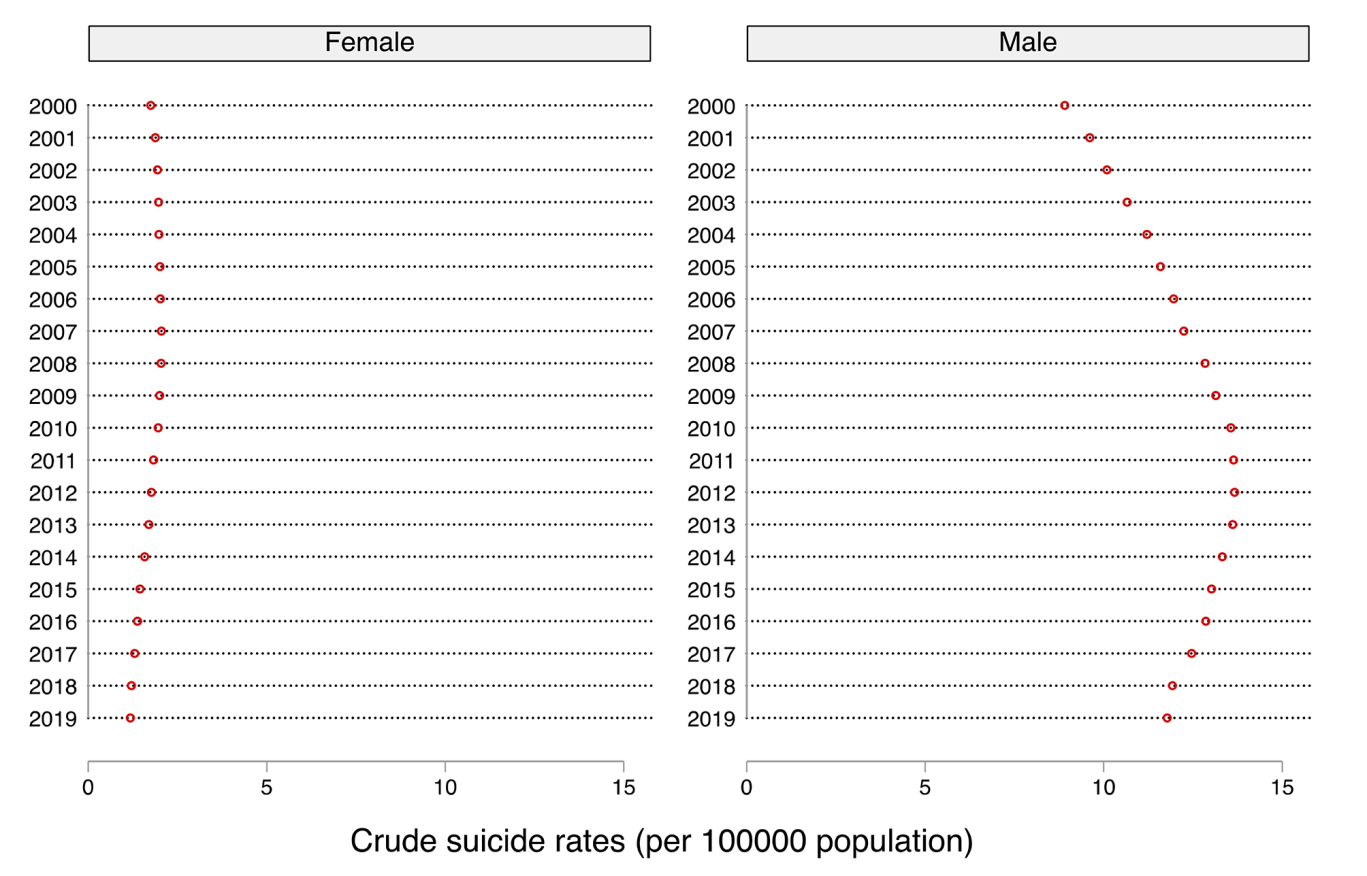
Sex differences in crude suicide rates in Ghana from 2000 to 2019

**Fig. 2:**
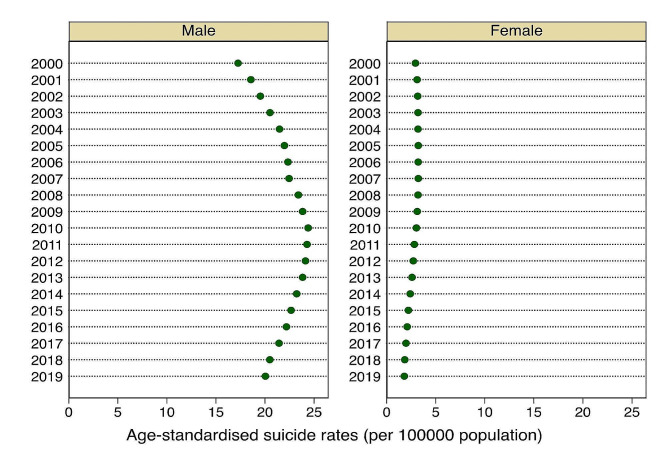
Sex differences in age-standardized suicide rates in Ghana from 2000 to2019

Figures 1 and 2 show the sex differences in crude and age-standardized suicide rates in Ghana from 2000 to 2019. Our findings revealed wide disparities in both age-standardized and crude suicide rates in Ghana. We observed varied trends of age-standardized and crude suicide rates for the men and women across the years surveyed. For instance, the age-standardized suicide rates among women showed a steady increase between the years 2000 and 2007, then declined marginally from 2008 to 2019, with the highest and lowest rates recorded in 2007 and 2019, respectively. Similar patterns were observed for crude suicide rates among the women. With regards to the men surveyed, the age-standardized suicide rates increased consistently from the years 2000 to 2010 before declining steadily from 2011 to 2019, with the highest and lowest rates recorded in the years 2010 and 2000, respectively. Similar patterns were observed for the crude suicide rates among the men.

**Fig. 3:**
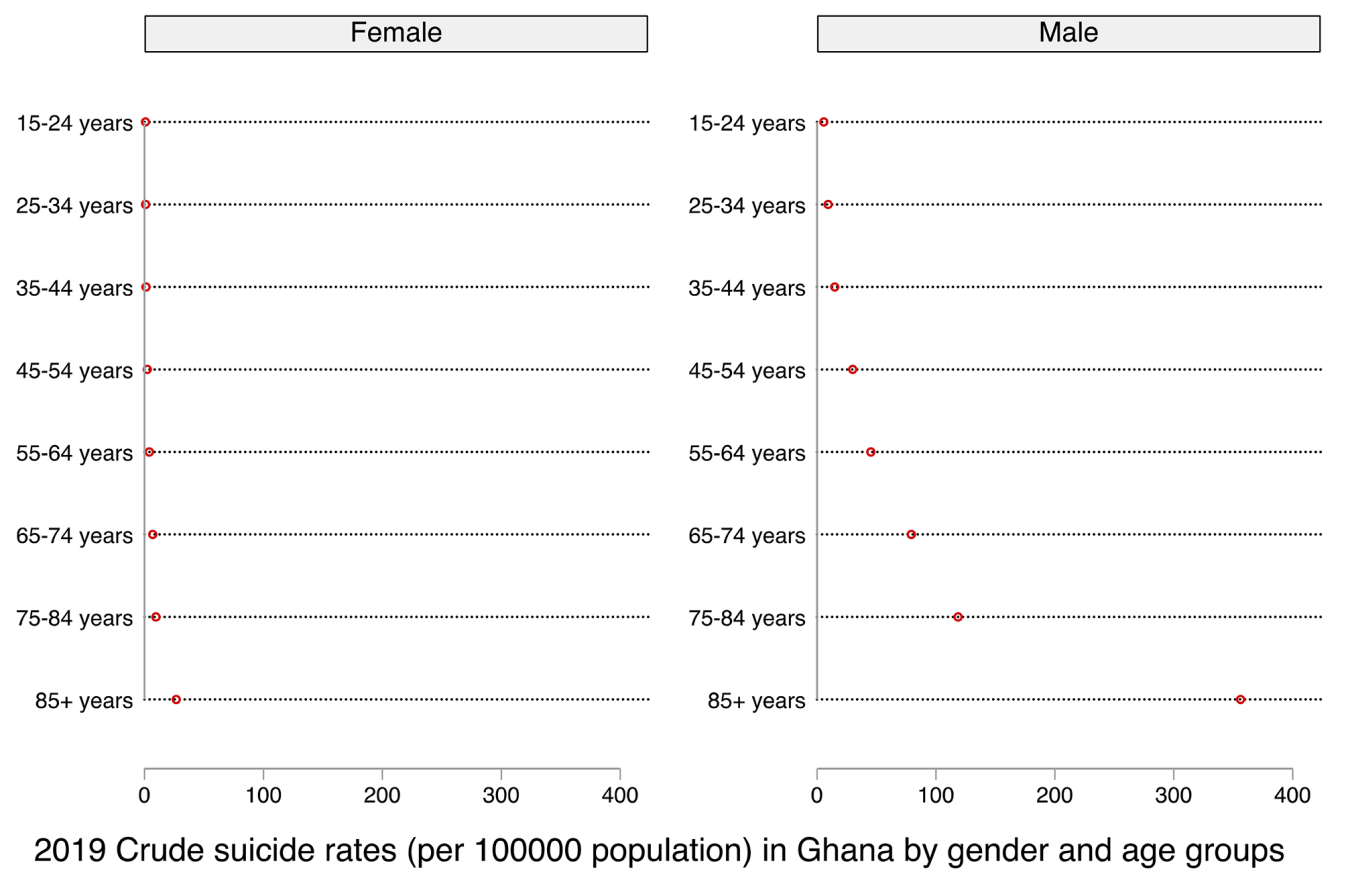
2019 Crude suicide rates (per 100,000 population) in Ghana by gender and age groups

Figure 3 shows the sex differences in crude suicide rates in Ghana among respondents aged 15–54 years and those aged 55–85+ yearsin 2019. For both age groups, crude suicide rates increased consistently with advancing age for both men and women. For example, among the respondents aged 15–54 years, the lowest crude suicide rates were observed among those aged 15–24 years while the highest rates were recorded among those aged 45–54 years. A similar trend was observed for both men and women aged 55–85+ years (Fig. 3). Therefore, for all the age groups, men and women aged 15–24 years had the lowest suicide rates, while those aged 85 years and above had the highest suicide rates. Thus, the rate of suicide increases with advancing age for both men and women, although wider increases were seen in men than women for all the age groups surveyed.

Tables 1 and 2 represent the sex-related inequalities in crude suicide rates and age-standardized suicide rates by summary measures, respectively. Both absolute (D) and relative (R) measures of inequalities showed substantial disparities in the rates of suicide, which disadvantaged men and the aged across all the years surveyed. For instance, inequalities in crude suicide rates show absolute (D) and relative (R) disparities that disadvantaged men in all the years surveyed (Table 1). The widest disparities in the absolute measures (D) of crude suicide rates were observed in 2013 (D= -11.91), 2012 (D= -11.90), 2011 (D= -11.81), and 2014 (D= -11.74), while the years 2000 (D= -7.16) and 2001 (D= -7.73) showed the least disparities. Similarly, the R values suggest that the rate of crude suicide rates is disproportionately higher among men in all the years surveyed, ranging from 0.20 in the years 2000 and 2001 to 0.10 between 2017 and 2019. The national estimates show that crude suicide rates increased from 2000 to 2010 and started decreasing between 2011 and 2019.

**Table 1:** Sex-related inequalities in crude suicide rates (per 100 000 population) by summary measures

Years	Difference (Estimate)	Ratio (Estimate)	Setting average
2019	-10.60	0.10	6.55
2018	-10.72	0.10	6.64
2017	-11.16	0.10	6.96
2016	-11.48	0.11	7.20
2015	-11.57	0.11	7.31
2014	-11.74	0.12	7.53
2013	-11.91	0.12	7.73
2012	-11.90	0.13	7.79
2011	-11.81	0.13	7.80
2010	-11.60	0.14	7.83
2009	-11.14	0.15	7.63
2008	-10.80	0.16	7.50
2007	-10.19	0.17	7.20
2006	-9.94	0.17	7.04
2005	-9.58	0.17	6.85
2004	-9.23	0.18	6.64
2003	-8.69	0.18	6.36
2002	-8.15	0.19	6.05
2001	-7.73	0.20	5.78
2000	-7.16	0.20	5.36

As shown in Table 2, the sex-related inequalities in age-standardized suicide rates revealed high absolute disparities in the rate of suicides across the ages of respondents, which disadvantaged the aged in all the years surveyed. We observed wider disparities in 2011 (D= -21.46), 2012 (D= -21.41), 2010 (D= -21.36), and 2013 (D= -11.25), with the least gaps seen in 2000 (D= -14.32) and 2001 (D= -15.46). Similarly, the R values suggest that the rate of age-standardized suicide rates is disproportionately higher among the aged men and women for all the years surveyed, ranging from 0.17 in the year 2000 and 2001 to 0.09 between 2016 and 2019. The national estimates show that crude suicide rates increased from 2000 to 2010 and started decreasing between 2011 and 2019.

**Table 2:** Sex-related inequalities in age-standardized suicide rates (per 100 000 population) by summary measures

Years	Difference (Estimate)	Ratio (Estimate)	Setting average
2019	-18.25	0.09	10.54
2018	-18.65	0.09	10.79
2017	-19.46	0.09	11.31
2016	-20.09	0.09	11.74
2015	-20.44	0.10	12.02
2014	-20.83	0.10	12.40
2013	-21.25	0.11	12.79
2012	-21.41	0.11	12.99
2011	-21.46	0.12	13.11
2010	-21.36	0.12	13.26
2009	-20.72	0.13	13.03
2008	-20.20	0.14	12.86
2007	-19.23	0.14	12.42
2006	-19.13	0.14	12.35
2005	-18.75	0.15	12.16
2004	-18.29	0.15	11.91
2003	-17.31	0.16	11.44
2002	-16.36	0.16	10.96
2001	-15.46	0.17	10.46
2000	-14.32	0.17	9.75

## Discussion

In this study, we found wide disparities in both age-standardized and crude suicide rates in Ghana, which skewed towards men and the aged. Both absolute and relative summary measures revealed substantial disparities in the rates of suicide, which disadvantaged men in all the years surveyed. While suicide rates among women increased minimally between 2000 and 2007 before declining steadily from 2008 to 2019, that of men showed wider increases between 2000 and 2010 before declining marginally from 2011 to 2019. The trend analyses also revealed that the rate of suicide increases with advancing age for both men and women, although wider increases were seen in men across the age groups.

Like the findings from previous studies in Ghana [20] and elsewhere [9, 53–56], we observed higher suicide rates (both age-standardized and crude) among men than women. The summary measures also revealed a disproportionately higher rate of suicide among men relative to the women surveyed. Several demographics [57], socioeconomic [58], and cultural [59] factors contribute to the increased rate of suicide among men. For instance, factors such as the breakdown in relationships, low socioeconomic status, job loss, low income, debt, poor social stability, and stringent sociocultural norms have been associated with suicidal deaths, particularly among men [60]. In most Ghanaian societies, men are socialized to be strong without seeking help when they are confronted with social challenges. Adinkrah [34] reported that most men who resort to suicide do so to deal with shame and dishonour which emanate from several predicaments including looming criminal prosecution, impotence, failure to fulfil economic and social responsibilities, loss of patriarchal control, and marital issues such as wife’s infidelity. For example, the sociocultural framework in Ghana mandates men to provide for the economic and material needs of the family [61], even in instances where the female partner has a better job and earns higher income [34]. Thus, being able to fend for one’s family is associated with the prestige of being a “real man”. Therefore, men who fail to meet these obligations are often seen as irresponsible and uncaring, which contributes to the feeling of guilt and shame leading to suicide [61]. Perhaps, our findings highlight the persistent need for targeted interventions to address the factors that contribute to the increased risk of suicide among men in Ghana.

Several previous studies have reported that older people, particularly those aged 65 years and above, have a higher incidence of suicide than any other age group [20, 62–64]. Similarly, our findings revealed that the rate of suicide was higher among the aged population relative to the younger ones in both sexes, although men showed greater increases in suicidal deaths with advancing age. In an analysis of media-reported incidents of suicides between 2005 and 2016 in Ghana, Adinkrah [20] found that most of the individuals who died by suicide were males, aged 60 to 65 years. The increased vulnerability of the aged to suicidal deaths has been associated with factors such as loneliness, grief over the death of a loved one, physical health problems like a chronic disease or pain [65], and cognitive impairment [66]. Although the extended family system in Ghana offers great support and protection to the aged [67], the increasing trend of urbanization and socioeconomic development has curtailed the traditional family care and protection for the aged [68]. Thus, many older people in Ghana are continuously exposed to neglect, abuse, and violent behaviours [69] which predispose them to an increased risk of suicide [20]. Meanwhile, despite the high rate of suicide among the aged population in Ghana, the bulk of media and public attention on suicide is limited to the youth and young adults [1], which contributes to the non-availability of interventions to prevent suicide among the older population [62].

Consistent with the declining trend of age-standardized suicide rates worldwide [9, 70], we observed that the age-standardized suicide rates in Ghana has declined steadily since 2008 and 2011 for women and men, respectively. However, available evidence suggests that the prevalence of age-standardized suicides in Ghana is higher than the global average, with the rate of decline also being slower than the global average [71]. The WHO argues that the present rate at which age-standardized suicides are declining is not enough to achieve SDG 3.4, which seeks to reduce by one-third all premature mortalities including suicidal deaths [9]. Thus, considering the current slow rate of decline of age-standardized suicides in Ghana, more stringent measures will be needed to address the menace of suicide in the country.

### Practical implications

Although our findings revealed a consistent decline in suicide rates in Ghana over the past decade, the rate of decline remains slower than the global average. Besides, the current prevalence of suicide in Ghana is still higher than the global average [71]. Considering that suicides are completely preventable, identifying the highly vulnerable population is key in designing and implementing targeted policy interventions to address the menace. Meanwhile, despite the high rate of suicide among the aged population in Ghana, the phenomenon is rarely given any attention. Thus, our findings highlight the need for urgent measures to minimize the risk of suicide among the older population in Ghana. Perhaps, such measures could be targeted at enhancing family and social support systems for the aged, especially the older men. Also, our findings provide important baseline data for monitoring suicide trends in Ghana, especially among men and the aged population. Besides, the findings could be used in guiding future studies on suicide in Ghana. For example, examining the main factors that predispose men and older people to suicide could be useful in designing and implementing targeted suicide intervention programmes to prevent or minimize the phenomenon in Ghana.

### Strengths and limitations

The main strength of our study is the use of a 20-year nationally representative data on suicide in Ghana, which enhances the generalizability of our findings. Additionally, our findings could be useful not only in the monitoring of suicide trends in Ghana, but in guiding future studies on suicides, particular among men and the aged population. Despite these strengths, some inherent limitations in this study must be acknowledged. First, we could not report on the methods used in committing suicide among the victims, since such data was not available in the dataset. Meanwhile, understanding the suicide rate by a method is also useful in developing interventions to address the phenomenon [56]. Second, aside from age and sex, no other co-variate was used in the analysis, thus, limiting the interpretation of our findings. Finally, the suicide data used in the analysis were not disaggregated by region or geographical location. Such geographical segregations are important not only in understanding the distribution of the problem but also in planning intervention programmes.

## Conclusion

The findings from this study highlight the increased rate of suicide among men and the aged population in Ghana. Thus, there is a need for urgent measures to minimize the risk of suicide among men and the older population in the country. Aside from guiding future studies on suicide, findings from this study could serve as important baseline data for monitoring suicide trends in Ghana, especially among men and the aged population. Knowing the most vulnerable individuals at risk of suicide and understanding suicide trends in Ghana is important in designing and implementing intervention programmes to minimize or prevent suicides in the country.

## Data Availability

The dataset used can be accessed at https://www.who.int/data/gho/data/themes/mental-health/suicide-rates.

## Abbreviations

GBD: Global Burden of Diseases
GHE: Global Health Estimates
GHO: Global Health Observatory
HEAT: Health Equity Assessment Toolkit
ICD: International Classification of Diseases
IHME: Institute of Health Metrics and Evaluation
LMICs: Low-and middle-income countries
SDG: Sustainable Development Goal
SSA: Sub-Saharan Africa
WHO: World Health Organization
